# Novel Criteria for When and How to Exit a COVID-19 Pandemic Lockdown

**DOI:** 10.3389/fdata.2020.00026

**Published:** 2020-07-24

**Authors:** Chenyu Li, Paola Romagnani, Hans-Joachim Anders

**Affiliations:** ^1^Medizinische Klinik und Poliklinik IV, Klinikum der Universität, Ludwig-Maximilians-Universität München, Munich, Germany; ^2^Excellence Centre for Research, Transfer and High Education for the Development of DE NOVO Therapies (M.E.M., P.R.), University of Florence, Florence, Italy

**Keywords:** COVID-19, SARS-CoV-2, SEIR epidemic model, basic reproduction number, lockdown measures

## Abstract

In the first month of 2020, severe acute respiratory syndrome coronavirus-2 (SARS-CoV-2), a novel coronavirus spreading quickly via human-to-human transmission, caused the coronavirus disease 2019 (COVID-19) pandemic. Italy installed a successful nationwide lockdown to mitigate the exponential increase of case numbers, as the basic reproduction number R0 reached 1 within 4 weeks. But is R0 really the relevant criterion as to whether or not community spreading is under control? In most parts of the world, testing largely focused on symptomatic cases, and we thus hypothesized that the true number of infected cases and relative testing capacity are better determinants to guide lockdown exit strategies. We employed the SEIR model to estimate the numbers of undocumented cases. As expected, the estimated numbers of all cases largely exceeded the reported ones in all Italian regions. Next, we used the numbers of reported and estimated cases per million of population and compared it with the respective numbers of tests. In Lombardy, as the most affected region, testing capacity per reported new case seemed between two and eight most of the time, but testing capacity per estimated new cases never reached four up to April 30. In contrast, Veneto‘s testing capacity per reported and estimated new cases were much less discrepant and were between four and 16 most of the time. As per April 30 also Marche, Lazio and other Italian regions arrived close to 16 ratio of test capacity per new estimated infection. Thus, the criterion to exit a lockdown should be decided at the level of the regions, based on the local testing capacity that should reach 16 times the estimated true number of newly infected cases as predicted.

## Introduction

In the first month of 2020, severe acute respiratory syndrome coronavirus-2 (SARS-CoV-2), a novel coronavirus spreading quickly via human-to-human transmission, caused the coronavirus disease 2019 (COVID-19) pandemic. In most countries, the disease started from few cases in one province or area and, depending on the efficacy of immediate containment measures, remained under control or lead to uncontrolled community transmission. In case early containment measures were not sufficient, the local outbreak turned into uncontrolled community transmission (Leung et al., 2020), ultimately addressed by social distancing and, in some cases, complete lockdown (Li C. et al., 2020). However, such mitigation measures come at large costs in terms of declining economic activity, employment rates, and wealth of a nation. Increasing depts, poverty, domestic violence, and mental health problems are only some of the economic and social consequences of such mitigation measures. In expectation of these trade-offs, when and how to install mitigation measures is a matter of debate among decision-makers. The same debate later occurred with regards to when and how one can implement the installed mitigation. Some countries installed different measures in each region depending on the extent to which COVID-19 affected the respective region. Not so for Italy.

In February 2020, Italy was the first country in Europe noting local outbreaks; these were in Veneto and Lombardy, two regions in the northeast and northwest of Italy, respectively, and, while early containment measures controlled the problem in Veneto, the infection spread in an uncontrolled manner in Lombardy. On March 8, the Italian government installed a nationwide lockdown during a moment where symptomatic COVID-19 was highly prevalent in Lombardy, while many other regions of Italy had seen few cases. This offers the unique possibility of analyzing the effect of identical mitigation measures on different phases of community spreading of COVID-19 using real world data.

## Materials and Methods

### Data Source

The data of tested, confirmed, hospitalized, and deceased cases of SARS-CoV-2 reported by provinces in Italy were obtained from the Italian Ministry of Health (Ministero della Salute, http://www.salute.gov.it/portale/nuovocoronavirus/homeNuovoCoronavirus.jsp?).

### Susceptible Exposed Infectious Recovered Model

We proposed a deterministic “Susceptible-Exposed-Infectious-Recovered” (SEIR) compartmental model based on the clinical disease severity and intervention measures. For the modified SEIR model, the population under consideration was stratified by six groups as susceptible (S), exposed (E), mild infectious (I), hospitalized (H), recovered (R), and deceased (D) compartments.

$$\begin{array}{lll} \frac{dS\left(t\right)}{dt} & = & -\frac{\beta S\left(t\right)I\left(t\right)}{N}\\ \frac{dE\left(t\right)}{dt} & = & \frac{\beta S\left(t\right)I\left(t\right)}{N}-\alpha E\left(t\right)\\ \frac{dI\left(t\right)}{dt} & = & \alpha E\left(t\right)-\left(\gamma +p\right)\;I\\ \frac{dH\left(t\right)}{dt} & = & pI\left(t\right)-\left(\gamma_{h}+\mu \right)\;H\left(t\right)\\ \frac{dR\left(t\right)}{dt} & = & \gamma I\left(t\right)+\;\gamma_{h}H\left(t\right)\\ \frac{dD\left(t\right)}{dt} & = & \mu H\left(t\right) \end{array}$$

The model was parameterized by using data obtained for the previous report of SARS-CoV-2, where β is the force of infection or disease transmission rate, α is the inverse of the latent period (days), (γ + *p*) is the inverse of the mild infectious period (days) or removal rate, *p* is the rate of mild cases progress to severe cases requiring hospitalization, (γ_*h*_ + μ) is the removal rate from hospitalization, and μ is the mortality rate for SARS-CoV-2 inpatient. Parameters are summarized in Table 1.

**Table 1:** Parameters of the susceptible-exposed-infected-removed model.

**Quantity**	**Parameter**	**Value**	**Source**
Basic reproduction number	R0	2.2 (1.6–3.0)	Kucharski et al., 2020; Li Q. et al., 2020; Wu et al., 2020; Zhao et al., 2020
Average incubation period	$\frac{1}{\alpha }$	5 days	Lauer et al., 2020
Average duration of mild infection	$\frac{1}{\left(\gamma \;+\;p\right)}$	6 days	Prem et al., 2020
Proportion of severe infections	$\frac{p}{\left(\gamma \;+\;p\right)}$	15%	Wu and McGoogan, 2020
Average time from onset of symptoms to death	–	18 days	Verity et al., 2020
Average Duration of hospitalization	$\frac{1}{\left(\gamma_{h}\;+\;\mu \right)}$	12 days	[Average time from onset of symptoms to death]- [Average duration of mild infection]
Case fatality ratio	$\frac{Server\%\;\times \;\mu }{\left(\gamma_{h}\;+\;\mu \right)}$	2.2–3.3%	Bassetti et al., 2020; Russell et al., 2020; Verity et al., 2020; Wang et al., 2020

### Estimation of Infected Cases and Basic Reproductive Number

Instead of the number of SARS-CoV-2-positive individuals reported by authorities, often falsely referred to as “infected cases” because they mostly represent the capability and intensity of testing activity, we employed the numbers of deceased cases. They provide a more robust estimate of outbreak trends, especially when the number of infected individuals exceeds by far the number of those tested positive. To reversely estimate the number of infected cases based on deceased cases number, we used cubic spline with a smoothing parameter of 0.6 to reduce the data noise of deceased cases and then calculated the number of hospitalized cases at time t, $H\left(t\right)=\frac{D_{t+1}-D_{t}}{\mu }$, infected cases with mild symptom number at time t, $I\left(t\right)=\frac{H_{t+1}+\left(\gamma_{h}+\mu -1\right)H_{t}}{p}$, and new recovered cases number at time t, *R*_*new*_(*t*) = γ*I*_*t*_ + γ_*h*_*H*_*t*_. All together, the total number of infected cases estimates at time t is: $I^{total}\left(t\right)=\;I\left(t\right)+\;H\left(t\right)+D\left(t\right)+\overset{t}{\underset{i=1}{\sum }}R_{new}\left(i\right)$. The reported hospitalized cases number was also used to estimate infected cases number by same strategy.

We assumed that, during the early phase, before depletion of susceptible individuals, the curve of infected individuals should follow an exponential increase with basic reproductive number (R0) = 2.5 as previously reported (Hellewell et al., 2020; Zhao et al., 2020). Upon installment of mitigation measures, a real-time reproductive number (*Rt)* was calculated according to a Bayesian framework algorithm established by Thompson et al. (Thompson et al., 2019). The probability of occurrence of a case was expressed as

$$\begin{array}{l} P\left(I_{t-\tau },I_{t-\tau +1},\ldots ,I_{t}|I_{0},I_{1},\ldots ,I_{t-1},W_{s},R_{t}\right)\\ =\prod_{K=t-\tau }^{t}\frac{{\left(R_{t}\Lambda_{k}\left(W_{s}\right)\right)}^{I_{k}}exp\;\left(-R_{t}\Lambda_{k}\left(W_{s}\right)\right)}{I_{k}!} \end{array}$$

where Λ_*k*_ represents the number of total infected individuals at time k, τ (7 days) represents the length of the time window over which *Rt* is estimated, and *W*_*s*_ is the serial interval distribution. Then we used a gamma distribution prior and conjugating to the Poisson likelihood to obtain an analytical formulation of the posterior distribution of *Rt* (Thompson et al., 2019). In addition to estimate *Rt* based on the reported infected cases, we also performed calculations using decease-estimated infected cases.

All analyses were performed using R software (version 3.6.1). *EpiEstim* package was used to implement *Rt* algorithm (Thompson et al., 2019).

## Results

### Italy Lockdown

**Figure 1:**
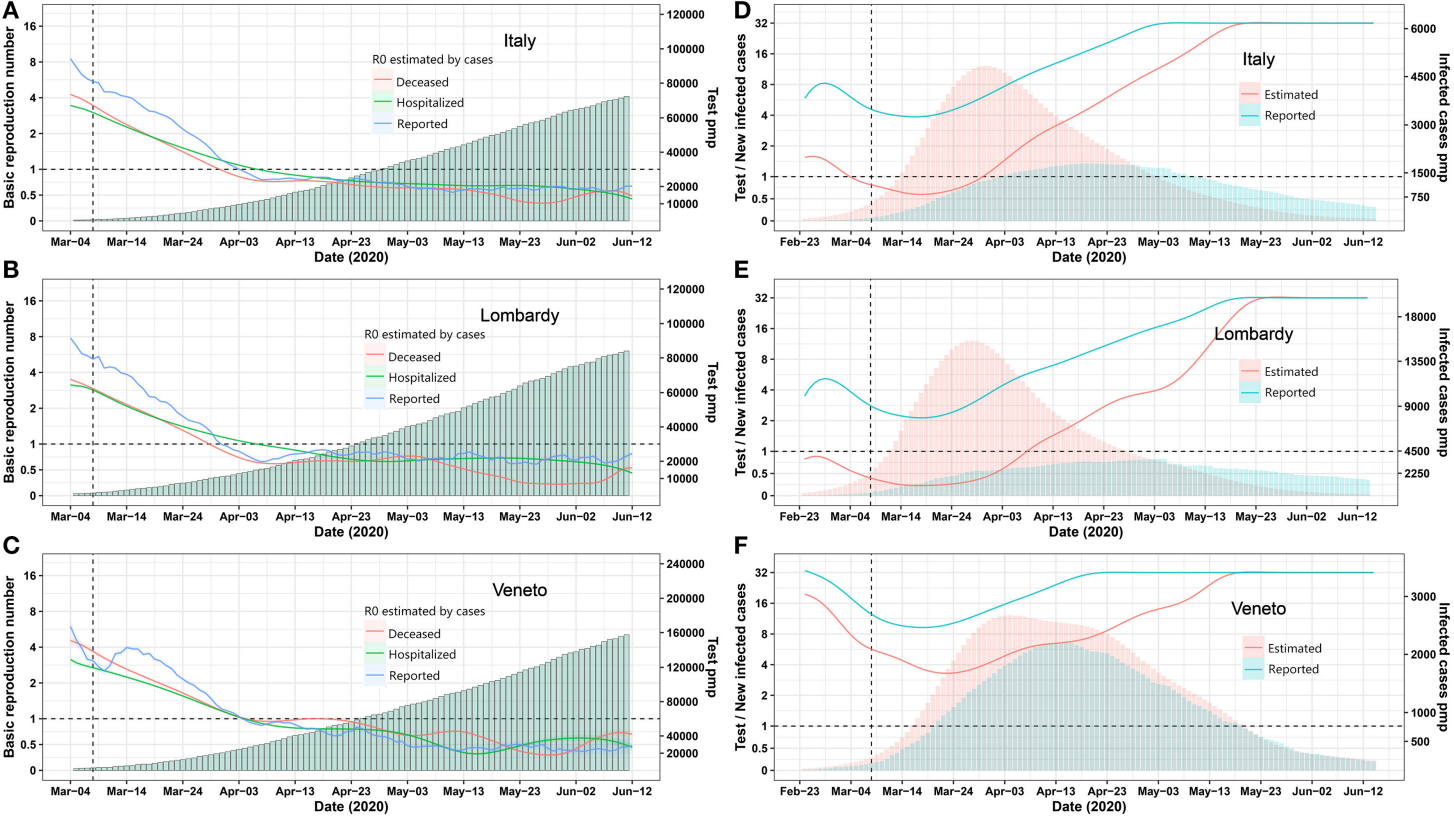
Epidemic trends for Italy and its provinces Lombardia and Veneto. **(A–C)** Real-time R0 and test per million. The blue, green, and red curve represents R0 estimated by using Bayesian framework algorithm based on reported, hospitalized, and deceased cases. The green bars represent test per million. **(D–F)** Daily test/estimated and reported infected cases. The green and red bars represent reported and estimated infected number. The green and red curves represent the number of daily test/reported and estimated infected cases. A vertical dashed line indicates the nationwide lockdown on March 8. Pmp, per million of population. All data were obtained from the Italian Ministry of Health (Ministero della Salute, http://www.salute.gov.it/portale/nuovocoronavirus/homeNuovoCoronavirus.jsp?).

On March 8, 2020, Italy installed a nationwide lockdown to mitigate the exponential increase of case numbers. We assessed its effect ex post by calculating the real-time R0 based on the reported tested positive cases and deceased cases to understand the dynamic changes of infection spreading. Above all, the Italian lockdown measures were successful, as the real-time basic reproduction number R0 for infected, hospitalized, and deceased cases were decreasing in a parallel manner and reached 1 on March 22, which meant the epidemic come under control. In most regions of Italy, the R0 declined to <1 within 4 weeks of lockdown (Figures 1A–C, Figure S1), but is R0 really the relevant criterion with which to determine whether or not community spreading is under control?

### Novel Criteria

Italy ramped up testing capacities to isolate infected individuals but again to a much different extent as per million of population in each region (Figures 1A–C, Figure S1). In most parts of the world, and thus most regions of Italy, testing largely focused on symptomatic cases, ignoring that the pandemic spreads via unrecognized asymptomatic individuals (Li R. et al., 2020). Therefore, we hypothesized that the true number of infected cases and relative testing capacity are better determinants to guide lockdown exit strategies and, because these parameters likely differ in each region, may suggest different exit strategies in each region.

We employed the “Susceptible-Exposed-Infectious-Recovered” (SEIR) model to estimate the numbers of all infected cases for each Italian region on the basis of reported deceased cases as these are more reliable (Figure S2). The prediction model was reliable, as predicted and reported numbers of hospitalized and deceased COVID-19 cases matched very well for most regions (Figure S2). As expected, the estimated numbers of all infected cases largely exceeded the reported ones in all regions (Tables S1, S2). Next, we used the numbers of reported and estimated cases per million of population and compared it with the respective numbers of tests (Figure 1D, Figure S3). In Lombardy, as the most affected region, testing capacity per reported new case seemed between two to eight most of the time, but testing capacity per estimated new cases never reached four up to April 30 (Figure 1E). In contrast, Veneto‘s testing capacity per reported and estimated new cases were much less discrepant and were between four and 16 most of the time (Figure 1F). As per April 30, Marche, Lazio, Campania, Puglia, Friuli Venezia, Giulia Sicilia, Umbria, Calabria, Basilicata, Liguria, and Veneto also arrived close to 16 ratio of test capacity per new estimated infection (Figure S3). Thus, the criterion to exit a lockdown should be decided at the level of the regions, based on the local testing capacity that should reach 16 times the estimated true number of newly infected cases as predicted.

## Discussion

The timing of reopening could be a complex and step-by-step issue, which needs to balance the local capacity to identify infected cases and the degree of social contact. Therefore, the question is how many people contact infected cases per day, and how many could get a test. The concept of testing/new cases is more like a parameter to assess the capacity for authorities to trace the potential cases exposed by one infected case. For example, the testing capacity is 16 times the new cases, which means 16 exposed cases get tested per newly infected case, and the number 16 is about equal to the number of people contacted per infected cases in lockdown setting. However, the number should be increased if we reopen since people have more chance to contact with others

On May 18, Italy reopened commercial activities—all regions' testing/new cases reached 16 ratios. Since this partial reopening, the epidemic remains under control without any subsequent adverse consequence, which supports our conclusion. With the continuous increase in testing capabilities, the number of infected cases is declining, and a full reopening is just around the corner.

A nationwide exit from lockdown would ignore that the capacity to control community spreading differs across regions, which is not sufficiently indicated by the basic reproduction number R0 (Hellewell et al., 2020). Thus, when and how to exit a lockdown should be decided at the level of the regions, or potentially even on a district level, based on the local testing capacity that should reach 16 times the estimated true number of newly infected cases as predicted, e.g., by the deceased cases in this district or region. Reaching congruency between estimated and documented cases and a sufficient capacity to isolate new cases are further requirements. Based on these indications, regions like, for example, Veneto, Campania, Friuli Venezia Giulia, Umbria, Calabria, Basilicata, or Sardegna may exit some of the lockdown measures earlier than Lombardy, Emilia-Romagna, or Piemonte if travel restrictions across the regions remain in place.

We believe there are not enough data to draw relevant conclusion about the consequence of a region being reopened before certain criteria are met, while, in our opinion, a test capacity of 16 ratios per new estimated infection is a robust criterion for the authorities to consider further strategies of exiting lockdown gradually. This model can help in making political decision also in other countries or regions of the world, provided that the necessary data are available at the regional or district level.

## Data Availability Statement

The original contributions presented in the study are included in the article/Supplementary Material, further inquiries can be directed to the corresponding author.

## Author Contributions

CL, PR, and H-JA conceived and designed the study. CL performed the statistical analysis and wrote the paper. PR and H-JA reviewed and edited the manuscript. All authors read and approved the manuscript.

## Conflict of Interest

The authors declare that the research was conducted in the absence of any commercial or financial relationships that could be construed as a potential conflict of interest.
