# Supplementary material for: Novel Criteria for When and How to Exit a COVID-19 Pandemic Lockdown
Source: Front Big Data. 2020 Jul 24;3:26. doi: 10.3389/fdata.2020.00026 (PMC7931856; doi:10.3389/fdata.2020.00026)
Supplement: Supplementary file 1 [file Data_Sheet_1.docx]

**Supplementary Appendix**

Supplement to Chenyu Li, Paola Romagnani and Hans-Joachim Anders.

Novel criteria for when and how to exit a COVID-19 pandemic lockdown

**Table of contents:**

| Supplemental Table S1 | Page 2 |
| --- | --- |
| Supplemental Table S2 | Page 3 |
| Supplemental Figure S1 | Page 4-5 |
| Supplemental Figure S2 | Page 6-7 |
| Supplemental Figure S3 | Page 8-9 |
| Acknowledgements | Page 9 |

**Correspondence:** Dr. Hans-Joachim Anders, Medizinische Klinik und Poliklinik IV, Klinikum der Universität, 80336 München, Germany. Email: hjanders@med.uni-muenchen.de

**Supplemental Table S1. Estimated and reported cases at March 8**

| **Region** | **R0 estimated by** | | |  | **Estimated cases at March 8** | | |  | **Reported cases at March 8** | | |
| --- | --- | --- | --- | --- | --- | --- | --- | --- | --- | --- | --- |
|  | **Reported cases** | **Hospitalized cases** | **Deceased cases** |  | **Isolated*** | **Hospitalized** | **Total infected** |  | **Isolated** | **Hospitalized** | **Total infected** |
| **Italy** | 5.48 | 3.72 | 4.13 |  | 19421 (18690,23336) | 1331 (1457,1480) | 20751 (20147,24816) |  | 2180 | 4207 | 6387 |
| **Lombardia** | 5.18 | 3.44 | 3.87 |  | 12012 (11344,15961) | 813 (872,1001) | 12824 (12216,16962) |  | 756 | 2616 | 3372 |
| **Emilia-Romagna** | 5.20 | 3.34 | 4.34 |  | 3344 (3031,4269) | 235 (233,278) | 3579 (3263,4547) |  | 480 | 617 | 1097 |
| **Piemonte** | 13.65 | 4.12 | 3.90 |  | 865 (840,1021) | 58 (62,62) | 922 (902,1082) |  | 65 | 290 | 355 |
| **Veneto** | 3.07 | 3.84 | 3.48 |  | 689 (676,871) | 49 (53,57) | 738 (730,928) |  | 430 | 193 | 623 |
| **Toscana** | 15.58 | 4.70 | 3.95 |  | 274 (248,376) | 19 (19,24) | 293 (267,400) |  | 67 | 98 | 165 |
| **Liguria** | 3.02 | 5.55 | 4.55 |  | 658 (640,900) | 48 (51,61) | 706 (691,961) |  | 17 | 50 | 67 |
| **Marche** | 13.21 | 4.12 | 5.38 |  | 769 (639,1024) | 60 (55,74) | 830 (695,1098) |  | 114 | 151 | 265 |
| **Lazio** | 12.75 | 4.41 | 3.81 |  | 166 (171,209) | 13 (14,15) | 178 (186,224) |  | 26 | 55 | 81 |
| **Campania** | 8.87 | 4.46 | 3.95 |  | 110 (76,203) | 8 (6,14) | 118 (82,217) |  | 63 | 37 | 100 |
| **P.A. Trento** | 27.87 | 3.91 | 4.36 |  | 73 (53,130) | 5 (4,9) | 79 (57,139) |  | 14 | 9 | 23 |
| **Puglia** | 12.94 | 4.27 | 3.86 |  | 126 (116,160) | 9 (10,11) | 135 (126,171) |  | 16 | 20 | 36 |
| **Friuli Venezia Giulia** | 14.11 | 3.92 | 3.72 |  | 284 (227,429) | 21 (19,30) | 306 (245,458) |  | 45 | 8 | 53 |
| **Sicilia** | 8.73 | 4.92 | 1.51 |  | 110 (76,203) | 8 (6,14) | 118 (82,217) |  | 33 | 18 | 51 |
| **Abruzzo** | 6.20 | 5.18 | 4.02 |  | 84 (60,151) | 6 (5,10) | 90 (65,161) |  | 3 | 14 | 17 |
| **P.A. Bolzano** | 8.64 | 4.67 | 3.89 |  | 96 (67,175) | 7 (6,12) | 103 (73,187) |  | 1 | 8 | 9 |
| **Umbria** | 14.53 | 4.26 | 2.08 |  | 32 (26,53) | 2 (2,4) | 35 (28,57) |  | 22 | 4 | 26 |
| **Sardegna** | 29.47 | 4.13 | 4.27 |  | 22 (18,34) | 2 (1,2) | 23 (19,36) |  | 6 | 5 | 11 |
| **Calabria** | 10.80 | 3.84 | 1.55 |  | 49 (37,83) | 4 (3,6) | 52 (40,89) |  | 4 | 5 | 9 |
| **Valle d'Aosta** | 40.71 | 3.94 | 1.56 |  | 96 (67,175) | 7 (6,12) | 103 (73,187) |  | 8 | 1 | 9 |
| **Basilicata** | 14.53 | 4.09 | 1.47 |  | 25 (20,39) | 2 (2,3) | 27 (22,42) |  | 2 | 2 | 4 |
| **Molise** | 21.45 | 2.82 | 2.20 |  | 25 (20,39) | 2 (2,3) | 27 (22,42) |  | 8 | 6 | 14 |

* Estimated isolated cases is the sum of exposed cases and mild symptoms cases calculated by Susceptible-Exposed-Infectious-Recovered model.

**Supplemental Table S2. Estimated and reported cases at April 30**

| **Region** | **R0 estimated by** | | |  | **Estimated cases at April 30** | | |  | **Reported cases at April 30** | | |
| --- | --- | --- | --- | --- | --- | --- | --- | --- | --- | --- | --- |
|  | **Reported cases** | **Hospitalized cases** | **Deceased cases** |  | **Isolated*** | **Hospitalized** | **Total infected** |  | **Isolation** | **hospitalized** | **Total infection** |
| **Italy** | 0.74 | 0.71 | 0.52 |  | 73099 (52961,74779) | 28030 (20341,30197) | 101129 (73302,104976) |  | 81708 | 18149 | 101551 |
| **Lombardia** | 0.83 | 0.57 | 0.31 |  | 26293 (17099,29286) | 11565 (7798,13823) | 37858 (24898,43109) |  | 28772 | 6834 | 36211 |
| **Emilia-Romagna** | 0.75 | 0.71 | 0.52 |  | 9783 (6776,10692) | 3744 (2679,4253) | 13527 (9455,14946) |  | 7211 | 2146 | 9563 |
| **Piemonte** | 0.79 | 0.80 | 0.78 |  | 12969 (8390,14217) | 3989 (2743,4672) | 16958 (11133,18889) |  | 12673 | 2621 | 15493 |
| **Veneto** | 0.61 | 0.79 | 0.98 |  | 4613 (3474,5626) | 1692 (1293,2099) | 6305 (4766,7725) |  | 7021 | 1012 | 8147 |
| **Toscana** | 0.54 | 0.63 | 0.74 |  | 3174 (2018,4148) | 981 (644,1325) | 4155 (2662,5473) |  | 4912 | 546 | 5584 |
| **Liguria** | 0.95 | 0.78 | 0.28 |  | 1854 (1207,2203) | 1171 (821,1501) | 3025 (2028,3704) |  | 2806 | 676 | 3551 |
| **Marche** | 0.60 | 0.65 | 0.29 |  | 1340 (770,1525) | 778 (484,960) | 2118 (1254,2485) |  | 2697 | 465 | 3210 |
| **Lazio** | 0.70 | 0.99 | 1.61 |  | 1779 (1339,2027) | 649 (512,776) | 2427 (1852,2803) |  | 2936 | 1417 | 4468 |
| **Campania** | 0.53 | 0.86 | 0.38 |  | 1016 (655,1274) | 441 (303,590) | 1457 (958,1865) |  | 2260 | 484 | 2773 |
| **Puglia** | 0.81 | 0.57 | 0.35 |  | 922 (580,1159) | 446 (301,601) | 1368 (881,1761) |  | 1191 | 158 | 1370 |
| **P.A. Trento** | 0.66 | 0.75 | 0.29 |  | 1158 (675,1310) | 528 (331,642) | 1686 (1006,1952) |  | 2468 | 439 | 2949 |
| **Friuli Venezia Giulia** | 0.64 | 0.86 | 1.05 |  | 793 (602,921) | 294 (228,349) | 1087 (830,1270) |  | 1050 | 109 | 1170 |
| **Sicilia** | 0.76 | 0.81 | 0.36 |  | 770 (477,1049) | 349 (224,493) | 1119 (701,1541) |  | 1716 | 408 | 2157 |
| **Abruzzo** | 0.51 | 0.92 | 1.11 |  | 968 (516,1601) | 385 (207,644) | 1353 (723,2245) |  | 1593 | 306 | 1915 |
| **P.A. Bolzano** | 0.33 | 0.81 | 0.33 |  | 664 (363,976) | 317 (180,485) | 981 (543,1462) |  | 663 | 126 | 802 |
| **Umbria** | 0.88 | 0.66 | 1.02 |  | 37 (22,44) | 59 (38,77) | 96 (60,121) |  | 149 | 68 | 233 |
| **Sardegna** | 0.43 | 0.83 | 0.93 |  | 226 (123,299) | 155 (93,225) | 381 (216,524) |  | 641 | 87 | 744 |
| **Calabria** | 0.45 | 0.77 | 1.09 |  | 33 (17,42) | 77 (44,109) | 110 (61,151) |  | 629 | 105 | 740 |
| **Valle d'Aosta** | 0.22 | 0.76 | 1.43 |  | 38 (19,51) | 93 (51,138) | 131 (69,188) |  | 9 | 76 | 89 |
| **Basilicata** | 0.50 | 0.79 | 0.37 |  | 78 (46,103) | 38 (24,54) | 116 (70,157) |  | 139 | 49 | 192 |
| **Molise** | 0.54 | 0.58 | 0.30 |  | 32 (20,40) | 19 (13,25) | 51 (33,65) |  | 172 | 17 | 190 |

* Estimated isolated cases is the sum of exposed cases and mild symptoms cases calculated by Susceptible-Exposed-Infectious-Recovered model.


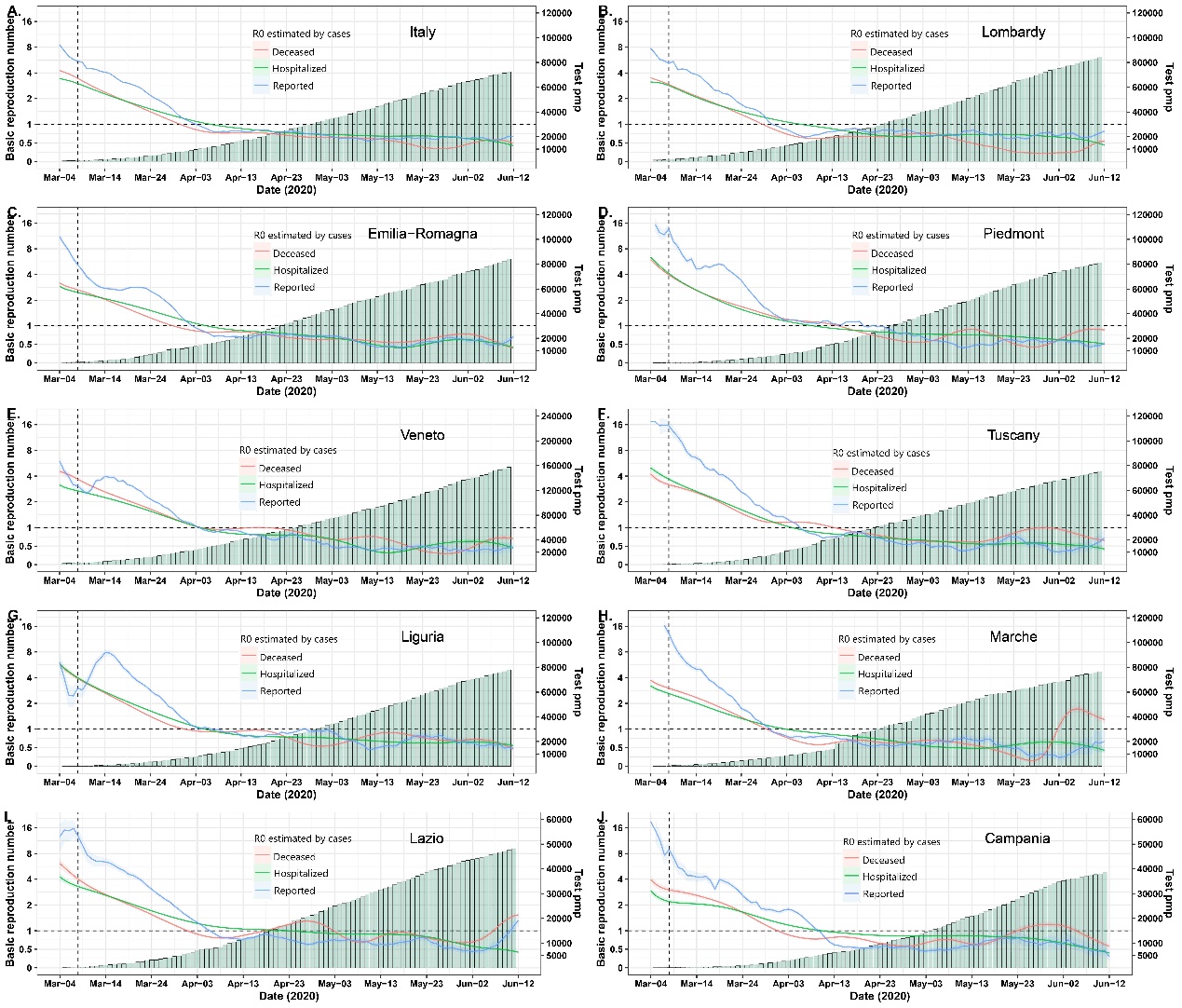

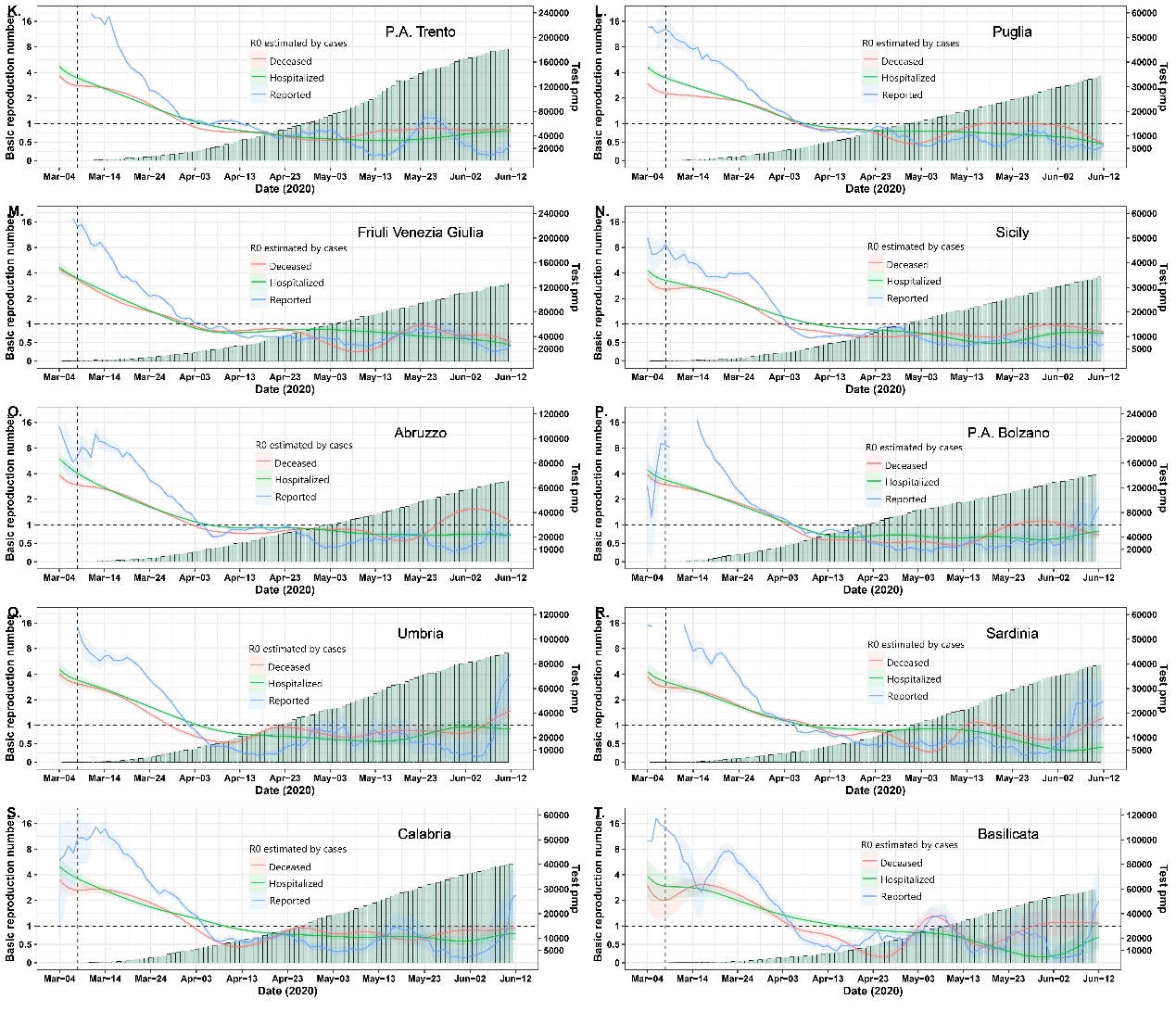


**Supplemental Figure S1.** **Real-time R0 and Test per million of population for all Italian regions.** The blue, green and red curve represents R0 estimated by using Bayesian framework algorithm based on reported, hospitalized and deceased cases. The green bars represents test per million A vertical dashed line indicates the nationwide lockdown on March 8. Pmp = per million of population. All data were obtained from the Italian Ministry of Health (Ministero della Salute,

<http://www.salute.gov.it/portale/nuovocoronavirus/homeNuovoCoronavirus.jsp>?).


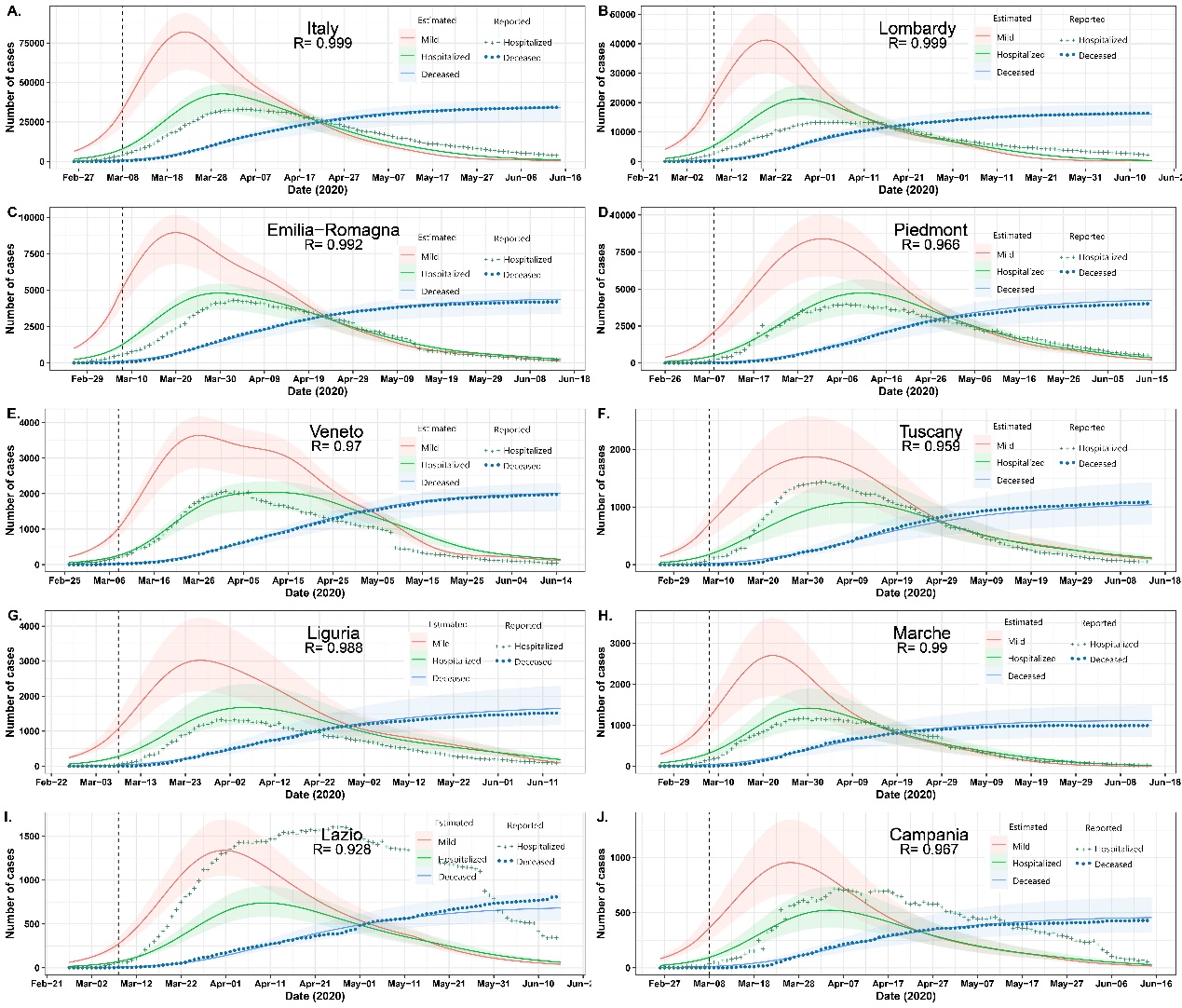

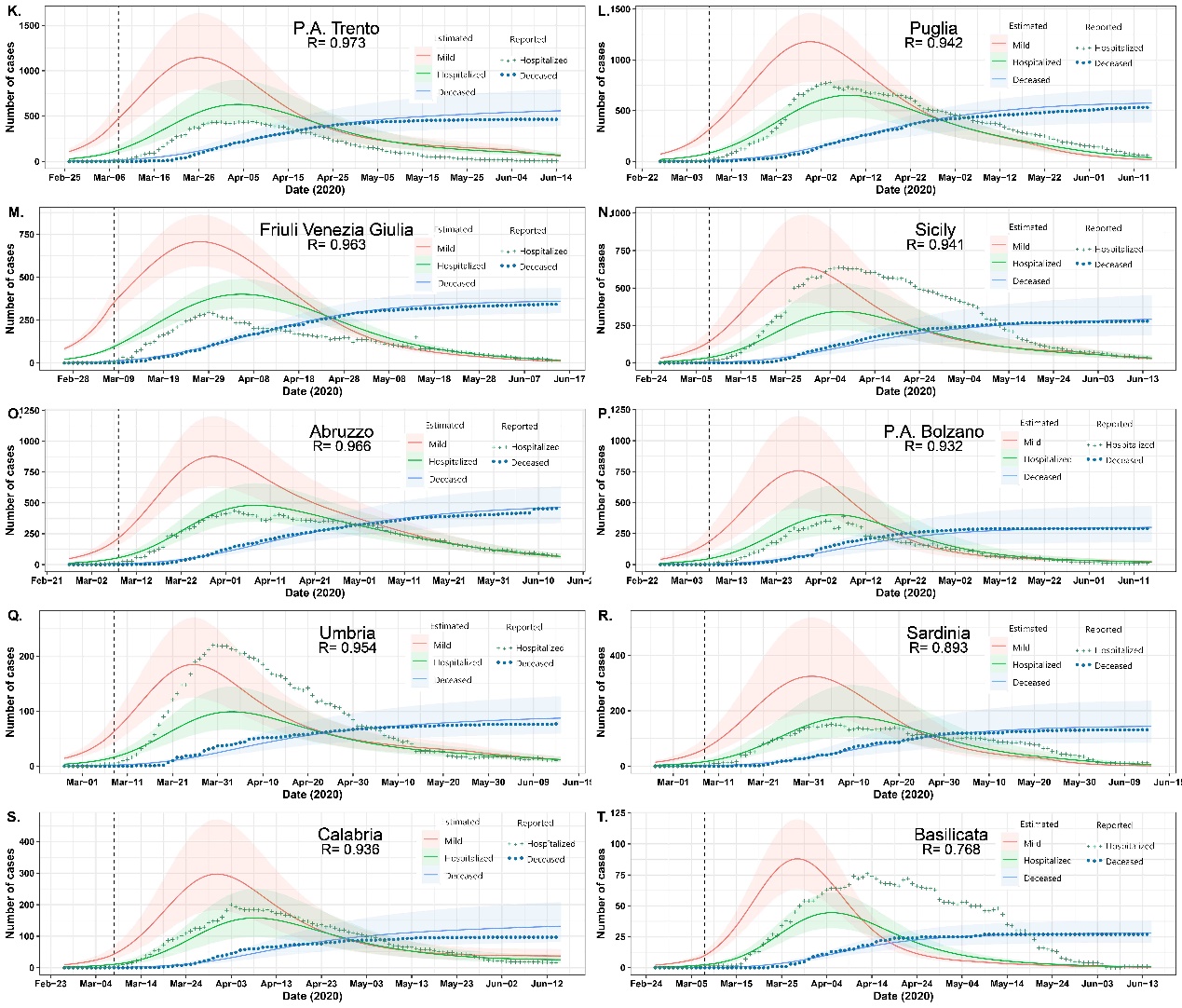


**Supplemental Figure S2. The Susceptible-Exposed-Infectious-Recovered model to estimate the infected, hospitalized and deceased cases.** The model was generated bases on reported deceased cases. All regions model showed high correlation coefficient between reported and predicted deceased cases. A vertical dashed line indicates the nationwide lockdown on March 8. All data were obtained from the Italian Ministry of Health (Ministero della Salute,

<http://www.salute.gov.it/portale/nuovocoronavirus/homeNuovoCoronavirus.jsp>?).


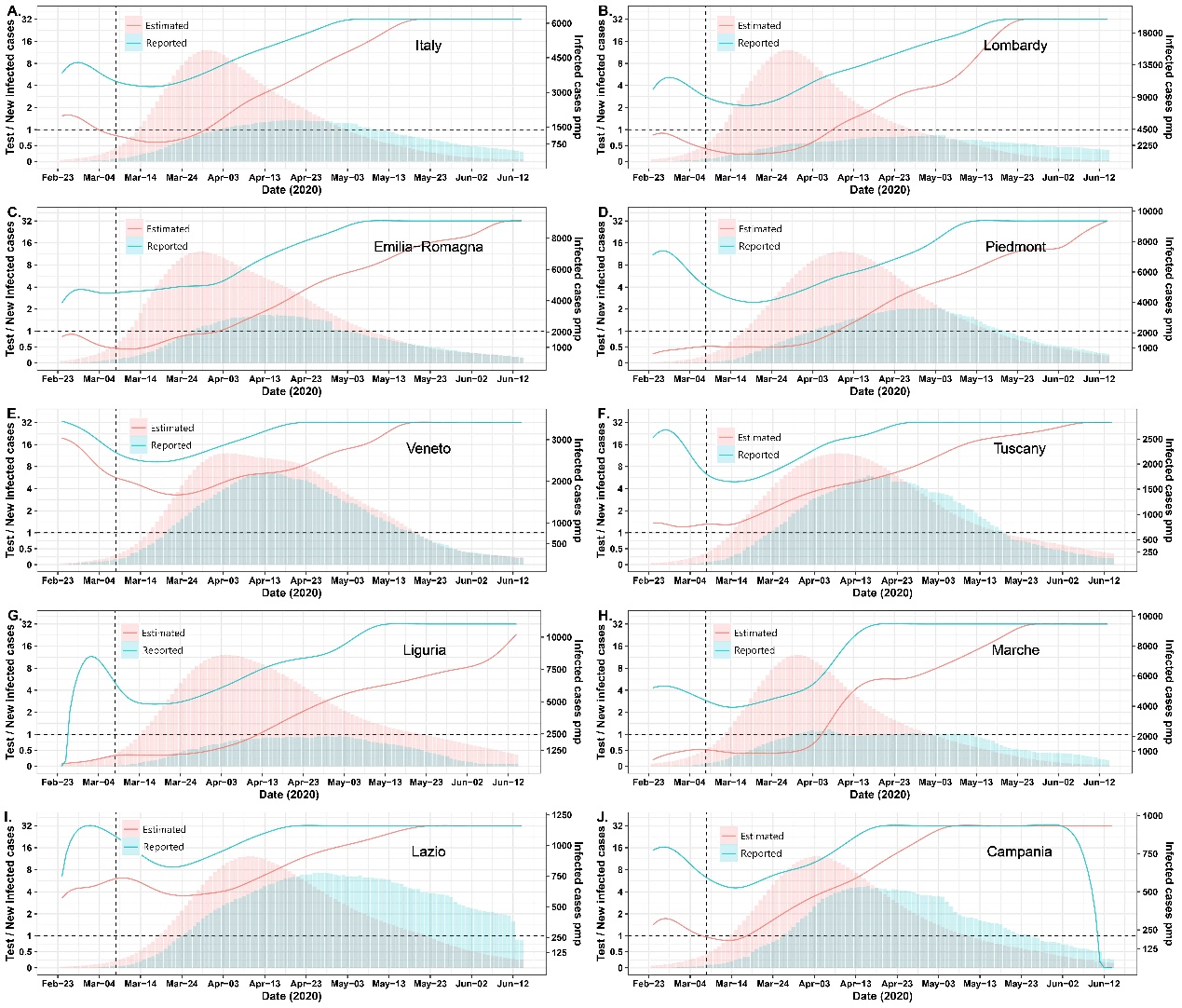

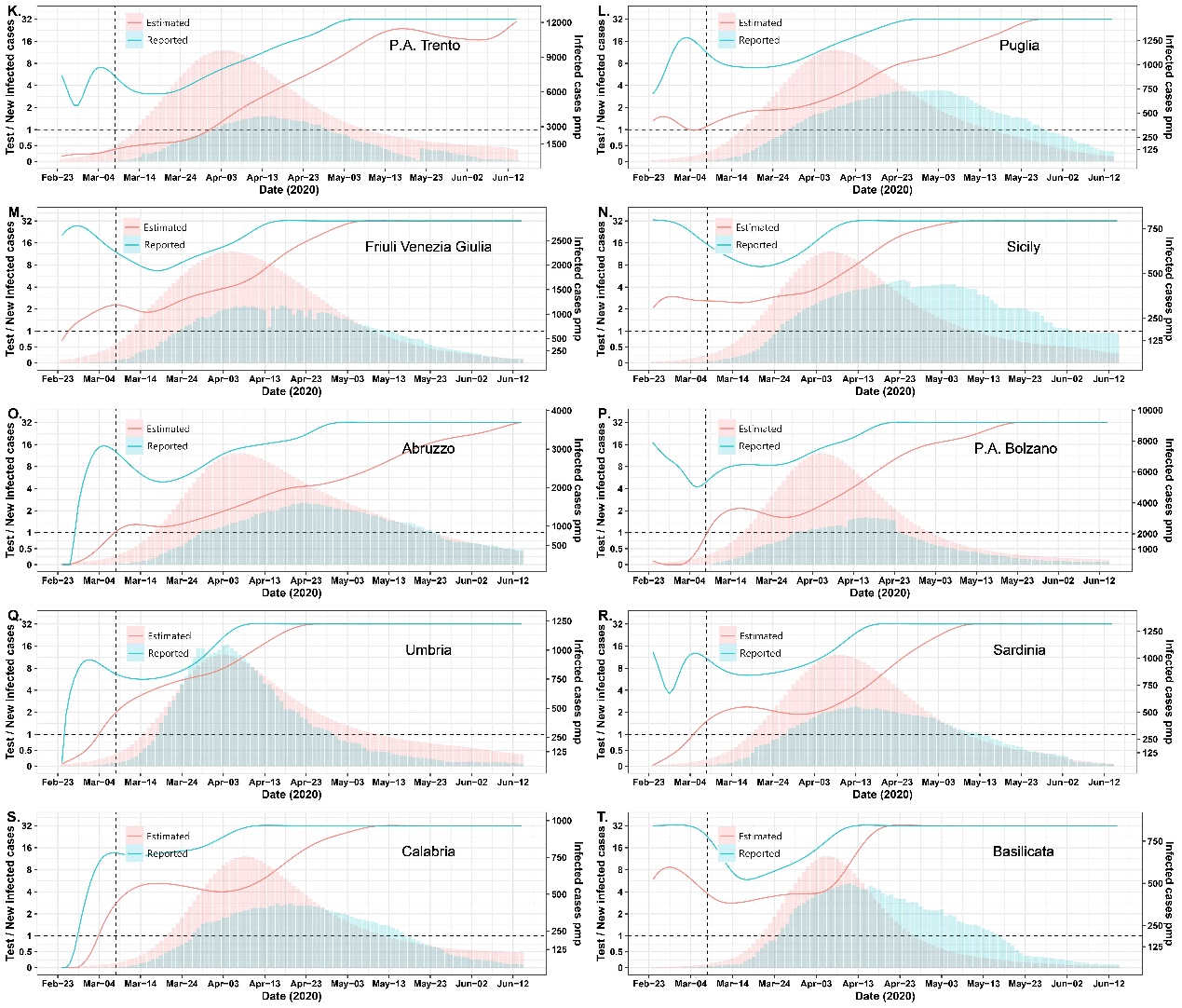


**Supplemental Figure S3.** **Test / new estimated and reported cases.** The green and red bars represents reported and estimated infected number. The green and red curve represents the number of daily test / reported and estimated infected cases. A vertical dashed line indicates the nationwide lockdown on March 8. Pmp = per million of population. All data were obtained from the Italian Ministry of Health (Ministero della Salute,

<http://www.salute.gov.it/portale/nuovocoronavirus/homeNuovoCoronavirus.jsp>?).

**Acknowledgements**

H.J.A. was supported by the Deutsche Forschungsgemeinschaft (AN372/24-1). No funding bodies had any role in study design, data collection and analysis, decision to publish, or preparation of the manuscript.
